# Supplementary material for: Stacking Model‐Based Classifiers for Dealing With Multiple Sets of Noisy Labels
Source: Biom J. 2025 Mar 12;67(2):e70042. doi: 10.1002/bimj.70042 (PMC11898607; doi:10.1002/bimj.70042)
Supplement: Supplementary file 1 — Supporting Information [file BIMJ-67-e70042-s001.zip › density-based-ensemble-model-main/competitors_real_data/netlab-master/netlab-master/help/linemin.htm]

Netlab Reference Manual linemin


# linemin

## Purpose

One dimensional minimization.

## Description

`[x, options] = linemin(f, pt, dir, fpt, options)` uses Brent's
algorithm to find the minimum of the function `f(x)` along the
line `dir` through the point `pt`. The function value at the
starting point is `fpt`. The point at which `f` has a local minimum
is returned as `x`. The function value at that point is returned
in `options(8)`.

`linemin(f, pt, dir, fpt, options, p1, p2, ...)` allows
additional arguments to be passed to `f()`.

The optional parameters have the following interpretations.

`options(1)` is set to 1 to display error values.

`options(2)` is a measure of the absolute precision required for the value
of `x` at the solution.

`options(3)` is a measure of the precision required of the objective
function at the solution. Both this and the previous condition must be
satisfied for termination.

`options(14)` is the maximum number of iterations; default 100.

## Examples

An example of the use of this function to find the minimum of a function
`f` in the direction `sd` can be found in `conjgrad`

```
x = linemin(f, xold, sd, fold, lineoptions);
```

## Algorithm

Brent's algorithm uses a mixture of quadratic interpolation and golden
section search to find the minimum of a function of a single variable once
it has been bracketed (which is done with `minbrack`). This is adapted
to minimize a function along a line.
This implementation
is based on that in Numerical Recipes.

## See Also

`conjgrad`, `minbrack`, `quasinew`

---

**Pages:**
Index


---

Copyright (c) Ian T Nabney (1996-9)
